# Leveraging Generative Artificial Intelligence Models in Patient Education on Inferior Vena Cava Filters

**DOI:** 10.3390/clinpract14040121

**Published:** 2024-07-30

**Authors:** Som P. Singh, Aleena Jamal, Farah Qureshi, Rohma Zaidi, Fawad Qureshi

**Affiliations:** 1 Department of Internal Medicine, University of Missouri Kansas City School of Medicine, Kansas City, MO 64108, USA; 2 Sidney Kimmel Medical College, Thomas Jefferson University, Philadelphia, PA 19107, USA; aleenakjamal@gmail.com; 3 Lake Erie College of Osteopathic Medicine, Erie, PA 16509, USA; 4 Department of Nephrology and Hypertension, Mayo Clinic Alix School of Medicine, Rochester, MN 55905, USA

**Keywords:** inferior vena cava filters, generative artificial intelligence, health literacy, patient education, readability

## Abstract

**Background**: Inferior Vena Cava (IVC) filters have become an advantageous treatment modality for patients with venous thromboembolism. As the use of these filters continues to grow, it is imperative for providers to appropriately educate patients in a comprehensive yet understandable manner. Likewise, generative artificial intelligence models are a growing tool in patient education, but there is little understanding of the readability of these tools on IVC filters. **Methods**: This study aimed to determine the Flesch Reading Ease (FRE), Flesch–Kincaid, and Gunning Fog readability of IVC Filter patient educational materials generated by these artificial intelligence models. **Results**: The ChatGPT cohort had the highest mean Gunning Fog score at 17.76 ± 1.62 and the lowest at 11.58 ± 1.55 among the Copilot cohort. The difference between groups for Flesch Reading Ease scores (*p* = 8.70408 × 10^−8^) was found to be statistically significant albeit with priori power found to be low at 0.392. **Conclusions**: The results of this study indicate that the answers generated by the Microsoft Copilot cohort offers a greater degree of readability compared to ChatGPT cohort regarding IVC filters. Nevertheless, the mean Flesch–Kincaid readability for both cohorts does not meet the recommended U.S. grade reading levels.

## 1. Introduction

Inferior vena cava (IVC) filters have long demonstrated clinical utility in improving patient outcomes regarding venous clot burden. They are designed to ensnare traveling clots, aiming to halt embolization within the pulmonary arterial system [1,2]. The clinical evidence on the use of IVC filters has continued to grow after demonstrating successful patient outcomes, especially among those with contraindications to anticoagulation [3,4]. Likewise, the array of IVC filter options provides interventional cardiologists, radiologists, and vascular surgeons with an advantageous treatment modality for their patients [5].

As the discussion on IVC filters continues to increase between patients and their providers, there is also a growing need to maintain the accuracy, understandability, and quality of the information available to patients regarding this treatment. It is well established in the literature that individuals will often utilize the Internet as a primary modality to acquire information regarding clinical diseases and therapies [6,7,8]. However, to some degree, there is no standardized regulation among sources of information available on the internet [9]. Regarding IVC filters, the literature suggests that the language used in online articles is considered difficult to read for the average population [10]. Additionally, there is a growing advancement in online information, which has recently experienced a surge in the use of generative artificial intelligence models to acquire information [11,12,13,14]. These models are developed based on a deep neural network that contributes to curating the response for the user [15]. Using generative artificial intelligence models may be advantageous for educating patients on numerous medical topics. Moreover, there is potential for clinicians to use generative artificial intelligence as a powerful tool to communicate with patients regarding their diseases or therapies.

For IVC filters, generative artificial intelligence may be able to explain to patients the risks, benefits, and uses of this treatment modality before a clinic visit and provide patients with greater control in understanding their clinical course as well as assist in curating questions for patients to prepare before seeing their providers [12]. This can allow clinicians to use generative artificial models as an educational resource for their patients. However, there remains a paucity of data that explores the quality and understandability of these responses. This study aims to explore the readability of generative artificial intelligence models in addressing questions asked by patients regarding IVC filters.

## 2. Materials and Methods

This study aimed to utilize publicly available generative artificial intelligence models as independent variables to evaluate the quality and utility of these tools in addressing the questions most frequently asked by patients. Similar to prior literature, this study employed the Google ^®^ RankBrain algorithm to generate 20 of the most frequently asked questions regarding IVC filters as demonstrated in Table 1 [16,17,18,19]. These questions were then extracted and applied to two generative artificial intelligence models—OpenAI ChatGPT and Microsoft Copilot [20,21]—to curate text responses to the questions. These responses were tabulated into a text document and qualitatively evaluated by the authors to determine whether the text responses provided by the generative artificial intelligence models relevantly answered the question. Additionally, the digital educational articles associated with each question by the RankBrain algorithm were extracted as equivalent text responses. This was selected as the control group, as it was the primary article aimed to answer the question by the Google RankBrain algorithm and not a generative artificial intelligence model answer.

The measurement outcomes of interest in this study were to evaluate the ease of comprehension of the answers provided by the generative artificial intelligence models. The comprehensibility was determined using Flesch Reading Ease, Flesch–Kincaid, and Gunning Fog readability calculations. These readability formulas are well established in the literature to evaluate the literacy capability of texts of interest [22,23,24,25,26,27]. Moreover, the Flesch Reading Ease and Flesch–Kincaid scores are calculated utilizing the total number of syllables, words, and sentences in the text of interest. The greater the Flesch Reading Ease score is, the higher it is associated with easier readability, whereas the Flesch–Kincaid score is associated with a U.S. educational grade (i.e., Flesch–Kincaid of 7.4 indicates a 7th grade reading level). Furthermore, the range of Flesh Reading Ease scores can be further elucidated into the following reading difficulty categories: ≤30 (very difficult to read); 31–50 (difficult to read); 51–60 (fairly difficult to read); 61–70 (standard difficulty to read); 71–80 (fairly easy to read); 81–90 (easy to read); ≥91 (very easy to read). While the Gunning Fog score also associates with a corresponding grade reading level like the Flesch–Kincaid score, its calculation also considers “complex” words, which are defined as words with more than three syllables. These readability formulas are demonstrated in Table 2.

## 3. Results

In February 2024, this study extracted 20 unique questions regarding the “Inferior Vena Cava Filter” from the Google RankBrain algorithm. Regarding the digital educational articles associated with each question, the articles were found to be most frequently sourced from an academic institution or commercial source at 35% each (*n* = 7). This was followed by sources from medical practices at 25% (*n* = 5) and from a government source at 5% (*n* = 1). Regarding the origin of each article, a majority of the articles were from the United States at 95% (*n* = 19) other than one article (*n* = 1).

Following extraction, a total of 180 readability scores were obtained from ChatGPT, Copilot, and the control groups (Table 1). Gunning Fog scores ranged from 8.2 to 15.0 for the control group, 14.3 to 20.9 for the ChatGPT, and 8.5 to 14.7 for the Copilot group. The mean Gunning Fog score was highest among the ChatGPT cohort at 17.76 ± 1.62 and the lowest at 11.58 ± 1.55 among the Copilot cohort. The control group score for Gunning Fog was 11.76 ± 2.56. This indicates that, on average, the Copilot cohort generated text responses equivalent to an 11th grade reading level in the United States, but the response generated to answer the same question would be equivalent to a postgraduate student level if the ChatGPT cohort was utilized.

The mean Flesch–Kincaid score was lowest among the control group at 9.65 ± 2.63 and the highest at 14.81 ± 1.37 among the ChatGPT cohort. The mean Flesch–Kincaid score for the Copilot cohort was 10.33 ± 1.46. These findings indicate that, on average, the responses generated by the Copilot cohort would be at the approximate reading level of a 10th grade student in the United States, whereas the text responses generated by the ChatGPT cohort would be at the approximate reading level of a college student.

**Figure 1:**
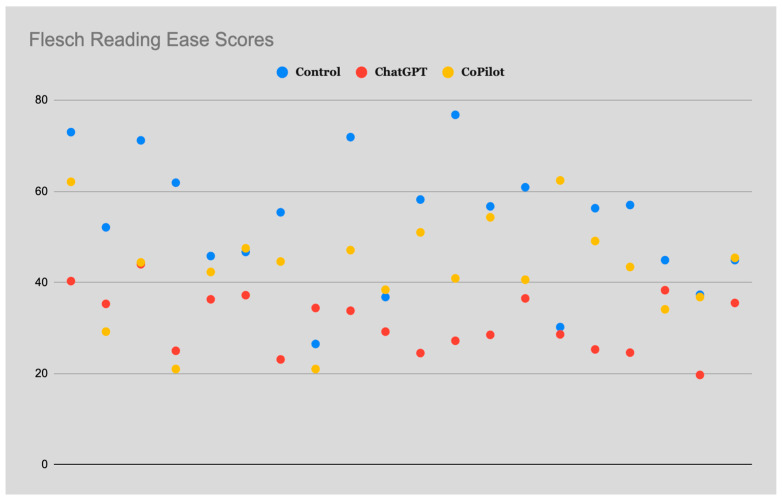
Distribution of Flesch Reading Ease Scores Among Generative Artificial Intelligence Models on IVC Filters.

The mean Flesch Reading Ease score was highest among the control group at 53.23 ± 14.18 and the lowest at 31.37 ± 6.62 among the ChatGPT cohort (Figure 1). The mean Flesch Reading Ease score for the Copilot cohort was 42.78 ± 11.06. These findings indicate that, on average, the text responses generated by both the ChatGPT and Copilot cohorts would be classified as “difficult to read” and equivalent to the grade reading level of a college student. The text responses generated by the control were, on average, considered to be “fairly difficult to read” and equivalent to the grade reading level range of a U.S. student in the 10th to 12th grade. Additionally, a statistically significant difference was found between the groups for the Flesh Reading Ease scores (*p* = 8.70408 × 10^−8^), with priori power found to be low at 0.392.

Regarding the source classification of the control group, the mean Gunning Fog score was 9.3 among the articles classified to be from medical practices (*n* = 5). The mean Gunning Fog was 11.77 for the articles from commercial sources (*n* = 7), and 12.46 for the articles from academic institutions (*n* = 7). The mean Flesch Reading Ease score was 65.64 among the articles classified to be from medical practices (*n* = 5). The mean Gunning Fog was 53.50 for articles from commercial sources (*n* = 7) and 49.69 for articles from academic institutions (*n* = 7). Government sources were not calculated as a mean (*n* = 1). 

## 4. Discussion

This study demonstrates the entry-level utility of publicly available artificial intelligence models to educate patients on inferior vena cava filters. This minimally invasive procedure by interventional radiologists and cardiologists has owned a degree of necessity as an effective and evidenced-based treatment option for venous thromboembolism [3,10]. However, appropriately disseminating this evidence to the public requires a nuanced level of interpretability so patients can understand the risks, benefits, indications, costs, and personal impact of this treatment option. There is a body of literature that has profiled various components of health literacy, including readability, which was a primary measurement outcome in this study [22]. Moreover, readability has a growing body of clinical literature, including on inferior vena cava filters, which have shown that most digital education articles need to meet the recommended U.S. reading level of materials, which is to be at the 6th grade level [22,28,29].

The mean Flesch Reading Ease score of the control group of this study would be interpreted as “fairly difficult to read” based on the prior literature. This finding aligns with the previous literature on inferior vena cava filters [10]. This further indicates that the current body of digital education on inferior vena cava filters carries a degree of difficulty, which may serve as a health literacy barrier for patients. Barriers to health literacy may translate to worse patient outcomes [17,30,31,32]. In this regard, barriers to health literacy on inferior vena cava filters may include loss of outpatient follow-up, filter retrievals, and filter indications. Thus, the inclusion of such barriers requires further investigation.

To the best of our knowledge, this study is the first to compare generative artificial intelligence tools in educating patients on inferior vena cava filters. A common denominator behind these models is that they are trained via large language models (LLMs) based on deep neural network architecture trained on language data [33,34]. The applied data on inferior vena cava filters form the basis of the curated responses generated by the artificial intelligence models to answer the questions asked. The public’s availability to use models such as ChatGPT and Copilot provides a growing avenue for disseminating medical information [35,36]. As this information is disseminated, it is imperative to maintain a degree of readability in the language models. The findings of this study suggest Microsoft Copilot provides a higher degree of readability than ChatGPT in all three measurement outcomes. This indicates that the answer provided by Copilot may be easier for patients to comprehend. However, the mean Flesch–Kincaid readability for both models does not meet the recommended U.S. grade reading levels. Moreover, it is difficult to determine the reliability of the significant difference found on ANOVA between groups for Flesch Reading Ease scores due to the priori power that has been found to be low at 0.392. The sample size of this study utilized 20 questions generated by Google RankBrain, but future investigations ought to aim to increase the sample size to evaluate statistical power of the findings. The decision to utilize 20 questions was because of a noted increase in irrelevant questions generated by the Google RankBrain on IVC filters after 20. Larger questions datasets may be generated by study authors or directly by patients who are exploring IVC filters. This makes it imperative to continue to evaluate generative artificial intelligence responses in order to truly determine if there is a significant difference in readability between using the generative artificial intelligence models.

To truly improve the health literacy of patients regarding this procedure, it is ever imperative to improve the readability of these generative artificial intelligence models as well. While the readability of patient education materials on IVC filters are not novel, a core strength of this study was that it is amongst the first studies to evaluate the current state of generative artificial intelligence on digital patient education on inferior vena cava filters. The findings provide preliminary data that can help improve these generative artificial intelligence models and provide more focused, readable answers to the questions extracted in this study. In addition, although the methodology provided in this study has been built off the previous literature [10,14,17,27,37], this study is one of the first to directly compare ChatGPT and Microsoft Copilot as generative artificial intelligence models. Future investigations can use this methodology to model studies regarding patient education on other endovascular procedures. Likewise, further comparing Copilot and ChatGPT among an array of endovascular procedures may provide further understanding regarding which model provides greater patient comprehension.

However, this study is not without its limitations. Two generative artificial intelligence models were utilized in this study. Still, the findings may not apply across generative artificial intelligence models in a general context given the caliber and degree of training each model undergoes. Moreover, this study quantitatively profiled the readability scores created by generative artificial intelligence models but the readability does not directly indicate the accuracy and quality of the text responses. These text responses were qualitatively evaluated for relevance by the study authors to ensure that the generated text responses directly answered the question about IVC filters. These findings can be built upon in future studies which utilize a quantitative assessment for quality, similar to readability. Potential quantitative tool may include utilizing DISCERN scores to establish a cutoff of a response making a threshold be considered as “good quality”.

While this study demonstrated the readability of text generated by these artificial intelligence models, it did not explore the utility to use these generative artificial intelligence models to generate simple text responses. Moreover, the findings of this study suggest that initial use of generative artificial intelligence does not provide education at the recommended grade reading level. Future investigation may attempt to use generative artificial intelligence to simplify the literature, possibly through entering command phrases such as “Explain this response at a 6th grade reading level”. This type of investigation could provide a greater understanding of generative artificial intelligence as patient education tool. Likewise, it is possible that these findings may not translate across the general patient population, given that there may be variable use of internet and digital resources such as generative artificial intelligence. Future investigations should analyze the statistical difference across various models to address this area better.

From the findings of both the control and generative artificial intelligence models, it is clear that internet-based educational content continues to require further evolution in order to be more readable. This may suggest that clinicians will need to spend a higher degree of time and resources to educate their patients on their questions as well as continued emphasis to their patients that generative artificial intelligence is not a physician nor equivalent to medical advice but rather a tool to be closely supplemented in addressing a patient’s curiosity at this time, similar to all internet resources. The publicly available models such as ChatGPT and Copilot are still considerably recent in their use so it is ever imperative that patients learn about them, since it is possible that this role may adapt over time as these models continue to train.

## 5. Conclusions

The findings of this study successfully leverage how generative artificial intelligence models can be used as educational resources to educate patients on inferior vena cava filters. Moreover, these models can be powerful tools for physicians to teach patients if further honed. While answer responses generated by Copilot demonstrate more excellent readability than ChatGPT, the current readability of the answers curated by these models often do not meet U.S. grade reading level recommendations. This makes it critical to enhance the quality of these responses to potentially improve patient health literacy of inferior vena cava filters. As a result, physicians currently educating patients on inferior vena cava filters should proactively explain the importance of where their information on inferior vena cava filters is taken from. Regardless, generative artificial intelligence models will continue to grow their digital footprint as tools in patient education.

## Figures and Tables

**Table 1:** Extracted questions regarding IVC filters.

Questions
What is the filter for the inferior vena cava?
What happens if IVC filter gets clogged?
Who should get an IVC filter?
When is it too late to remove IVC filter?
What are symptoms of IVC filter problems?
Can you still have a stroke with an IVC filter?
Can an IVC filter stay in permanently?
Can you still get a clot with an IVC filter?
What is the success rate of IVC filter?
Do you need blood thinner after IVC filter?
What to expect after IVC filter placement?
How do you fix a clogged IVC filter?
Should I have my IVC filter removed?
Can IVC filter cause pulmonary embolism?
How long does an IVC filter procedure take?
Why would someone need an IVC filter?
Is IVC filter removal a major surgery?
What happens if an IVC filter cannot be removed?
What is the most common IVC filter complication?

**Table 2:** Mean Gunning Fog, Flesch–Kincaid, and Flesch Reading Ease Scores Among Generative Artificial Intelligence Models on IVC Filters.

Readability	Formula
Flesch Reading Ease	206.835 − 1.015 (word count/sentence count) − 84.6 (syllable count/word count)
Flesch Kincaid	0.39 (word count/sentence count) + 11.8(syllable count/word count) − 15.59
Gunning Fog	0.4 [(words/sentences) + 100 (total number of words with ≥3 syllables/words)]

## Data Availability

The original contributions presented in the study are included in the article.
